# Biologically Inspired and Rehabilitation Robotics 2020

**DOI:** 10.1155/2022/9852938

**Published:** 2022-01-22

**Authors:** Liwei Shi, Yong Yu, Nan Xiao, Dongming Gan, Wei Wei

**Affiliations:** ^1^The Institute of Advanced Biomedical Engineering System, School of Life Science, Beijing Institute of Technology, No. 5, Zhongguancun South Street, Haidian District, 100081 Beijing, China; ^2^Kagoshima University, Kagoshima, Japan; ^3^Purdue University, West Lafayette, IN 47907, USA; ^4^Soochow University, Suzhou, China

Bioinspired methods are becoming increasingly important in the face of the complexity of today's demanding applications. Biological inspiration in robotics is leading to complex structures with sensory-motor coordination, in which learning often plays an important role in achieving adaptation. In addition, rehabilitation robotics has produced exciting new ideas and novel human assistive devices in the growing field of biomedical robotics. The science and technology of rehabilitation robotics will progress through the collaboration among robotic researchers, medical doctors, and patients.

This special issue focuses on the theoretical and technological challenges of evolutionary transformation from biological systems to intelligent robots. There were 14 original research papers that were finally accepted in this special issue after formal peer reviews. The accepted papers can be further classified into two related topics including rehabilitation and human assisting systems and bioinspired manipulator system for fine manipulation, surgery, robotics, and human-robot interaction applications.

Among the rehabilitation and human assisting systems, a big focus was on the lower limb and upper limb applications. H. Woo et al. investigated how climbing assistance by a robotic exoskeleton affects energy consumption. Despite lack of individual optimization, assistive joint torque applied to the hip and knee joints reduced metabolic cost and cardiovascular burden of stair climbing in healthy young males. The prediction of sensor data can help the exoskeleton control system to get the human motion intention and target position in advance, so as to reduce the human-machine interaction force. S. Zha et al. designed a human motion capture system to acquire human walking joint data and proposed a method for optimizing parameters of Takens' nonlinear prediction algorithm. Compared with the original Takens prediction algorithm, the prediction angle obtained by the improved prediction PSO-Takens algorithm was more closely related to the actual motion angle data of the human body, with a smaller error rate and smooth features. Robotic exoskeletons (RE) for motor rehabilitation can provide the user with consistent, symmetrical, goal-directed repetition of movement, and balance and stability. K. K. Karunakaran et al. evaluated the therapeutic effect of RE training on the loading/unloading and spatial temporal characteristics in adolescents and young adults with chronic ABI. Results showed improved step length, speed, and an overall progression towards healthy bilateral loading, with linearity of loading showing a significant therapeutic effect (*p* < 0.05). These results suggested that high dose, repetitive, consistent gait training using RE has the potential to induce recovery of function in adolescents and young adults diagnosed with ABI. K. Park et al. implemented the optimal design of a motorized prosthetic leg and evaluation of its performance for stair walking. Developed prosthetic leg includes two degrees of freedom on the knee and ankle joint designed using a virtual product development process for better stair walking. The DC motor system was introduced to imitate gait motion in the knee joint, and a spring system was applied at the ankle joint to create torque and flexion angle. The motorized prosthetic leg was optimally designed while maintaining structural safety under boundary conditions based on the human walking data, and its knee motions were synchronized with normal human gait via a PD controller. The results from this study might help amputees in their rehabilitation process and be applied to the area of biped robots that try to mimic human motion. The upper limb rehabilitation robots can be developed as an efficient tool for motor function assessments. Circle drawing has been used as a specific task for robot-based motor function measurement. C. Wang et al. used the robot-assisted and constrained circle drawing movements for upper limb to increase the consistency of muscle synergy features. In upper limb rehabilitation training by exploiting robotic devices, the qualitative or quantitative assessment of human active effort is conducive to altering the robot control parameters to offer the patients appropriate assistance, which is considered an effective rehabilitation strategy termed as assist-as-needed. Accordingly, a shoulder passive torque prediction method based on a backpropagation neural network (BPANN) was proposed to expand the shoulder passive torque-angle relationship by S. Li et al. Experiments were carried out to measure the kinematics and torques on the shoulder joint of 3 healthy subjects, and the measurement data was used as training set and testing set of a three-layer BPANN to test the prediction effect. Surface electromyography- (sEMG-) based hand grasp force estimation plays an important role with a promising accuracy in a laboratory environment, yet hardly clinically applicable because of physiological changes and other factors. J. Wang et al. proposed an easy-to-implement method to quantitatively estimate muscle fatigue and evaluated the effect of muscle fatigue on hand grasp force estimation. The experiment results demonstrated that the incorporation of muscle fatigue metrics explicitly in the grasp force estimation had a substantial impact on the performance.

Biology systems have showed good references and inspiration for engineers to develop new manipulation, robot system, and sensing systems. X. Ma et al. proposed a new catheter operating system of the surgical robot and designed a new mechanical structure of force feedback to measure the near-end force during the operation. A new controller, BP neural network PID controller, was designed by X. Ma et al., which could improve the axial and rotary motion accuracy of the system in remote operation. Simulation results show that the proposed BP neural network PID controller has good dynamic response quality. In addition, the robot of neurointerventional surgical has been continuously investigated and improved though 10 animal experiments. The robot system can realize the cooperative operation of the guidewire, and the catheter and has a force feedback system with good accuracy and stability. And various mechanical performance indexes basically meet the needs of vascular intervention surgery. J. Jiang et al. designed a passive ultrasound probe position and posture adjustment mechanism to assist doctors performing prostate scans and puncture interventions. In this paper, the forward kinematic analysis of the mechanism, the simulation of the centering effect, the development of the physical prototype, and related experimental research were presented.

Therefore, this special issue presents the most recent advances in modeling, design, analysis, control, implementation, and therapeutic testing of the human assistive rehabilitation systems, bioinspired prosthesis, manipulators, surgical robots, and sensing systems. We hope the knowledge and information will be good references and basis for further development in those fields for human centered science and technology.

*Liwei Shi* *Yong Yu* *Nan Xiao* *Dongming Gan* *Wei Wei*

